# Accuracy of CAD/CAM Technology in Fabricating Custom Post‐and‐Core Restorations: A Comparative Analysis

**DOI:** 10.1111/jerd.13438

**Published:** 2025-02-12

**Authors:** Petros Mourouzis, Dimitrios Dionysopoulos, Kosmas Tolidis

**Affiliations:** ^1^ Department of Dental Tissues Pathology and Therapeutics, Division of Operative Dentistry, Faculty of Dentistry Aristotle University of Thessaloniki Thessaloniki Greece

## Abstract

**Objective:**

This study aims to compare the accuracy of various intraoral scanners and traditional analog impressions in scanning root canals for CAD/CAM post‐fabrication.

**Methods:**

A dental mannequin with a factory‐made #21 tooth root canal was used to simulate clinical settings. Three intraoral scanners were tested: CEREC Primescan, TRIOS 3, and CEREC Omnicam. The inEos X5 desktop scanner provided high‐resolution control images. The analog impression polyvinyl siloxane (PVS) material was used with custom trays, following standard protocols. The surface areas were measured with CAD software and compared with the desktop scanner's reference.

**Results:**

Primescan demonstrated the highest accuracy among the intraoral scanners, followed by Trios 3 and Omnicam. Significant differences were found between each scanner and the true surface area. The analog impression method demonstrated superior accuracy compared with intraoral scanners; however, this high precision is attributed to irregularities in the silicone material.

**Conclusions:**

The analog impression method was more accurate in capturing the details of complex root canal anatomy; however, material irregularities can affect its clinical efficacy. Intraoral scanners showed high accuracy but had some limitations in capturing complex geometries. Further development of scanner technology will increase precision and, therefore, the clinical outcome.

**Clinical Significance:**

The use of CAD/CAM technology and intraoral scanners offers potential for precise, custom‐fit post‐and‐core restorations.

## 1 Introduction

Restoring severely damaged coronal structure of the endodontically treated teeth is a challenging task. Success depends upon a variety of factors, mostly partial or complete coverage restorations [1]. Custom cast posts and factory‐made fiber‐reinforced composite (FRC) posts are the two popularly used restorative options available for severely damaged endodontically treated teeth [2]. Repeated unsuccessful attempts, attributed to the markedly elevated metal posts' elastic modulus (up to 200 GPa, depending on the type of alloy used) in contrast to root dentin (18 GPa), have been observed to impact the distribution of stress within the dental architecture, consequently heightening the susceptibility to root fractures [3]. Additionally, in the cases of different root anatomy and in some of them an anatomy of funnel‐shaped root canal, the inadequacies of preformed fiber posts have become evident, as they are unable to conform optimally to the root cavity's contours [4]. Consequently, the adhesive interface emerges as a vulnerable juncture between dentin and the fiber post, leading to an elevated incidence of restoration failures like post debonding or crown dislodgements [2, 3]. Furthermore, the canal's anatomy presents an exceedingly unfavorable surface geometry for alleviating the shrinkage stresses that arise during resin cement polymerization [5].

Newer approaches for the restorations of endodontically treated teeth have suggested the use of endocrowns [6]. While the endocrown has been reported to exhibit a reasonable survival rate, some studies have not shown uniformly positive outcomes. Previous research has documented instances of root fractures, debonding, excessive preparation of the pulp chamber, and deviations from the minimally invasive approach associated with endocrown restorations. These findings suggest that the endocrown's inability to establish a connection with the root renders it prone to fracture, especially when the residual tooth tissue is compromised and forces are unevenly distributed [7]. Considerably reduced maximum von Mises (mvM) stresses have been observed in anterior teeth treated with posts and cores compared to those restored with endocrowns. Therefore, the restoration of incisors utilizing posts and prosthetic crowns continues to be more advisable than opting for endocrowns [8]. There are alternative solutions for the restoration of severely damaged endodontically treated teeth; this involves silanated glass fibers embedded in a thermoplastic polymer and light‐curing resin matrix (Everstick), composed of continuous unidirectional E‐glass fibers and a multiphase polymer matrix. This material offers high flexural strength once light‐cured and elasticity very similar to that of dentin and can be individually formable glass fiber root canal posts [9]. However, clinical studies during a 6‐year follow‐up period concluded that the incorporation of either a prefabricated or custom‐made post significantly enhanced the survival rates of endodontically treated teeth compared with glass fibers (success rate of 76.6% vs. 61.3%) [2].

The advancements in computer‐aided design and computer‐aided manufacturing (CAD/CAM) have evolved rapidly due to advancements in processor power. The clinical usage of CAD/CAM includes the design and fabrication of single crowns, bridges, inlays, onlays, veneers, implants, abutments, or screw‐retained implant crowns. In the first usage of CAD/CAM for the fabrication of a CAD/CAM post, an indirect technique was described, in which a custom zirconia post and core were crafted by milling a zirconia block. In this technique, an acrylic resin pattern recorded the post space anatomy, later scanned, milled, and sintered for the final ceramic post and core. Another method used auto‐polymerizing resin for pattern creation, then scanned and milled at a lab [10, 11]. However, in spite of the indirect technique, there is also a fully digital or direct technique in which the post space is captured directly from the intraoral scanner. Subsequently, a restoration design is generated using the design software, followed by milling. This direct technique of scanning the post space eliminates inaccuracies associated with impression materials, gypsum models, and pattern materials like resin and wax [12]. The advantage of CAD/CAM usage for the fabrication of posts is that it enables the production of post‐and‐core structures as a single unit, thereby minimizing the interfaces between the fiberglass post and resin composite core. This reduction in interfaces lowers the risk of material structural failure due to a meticulously controlled milling process of uniform material blocks. Custom‐made CAD/CAM post‐and‐core systems, manufactured through a precisely regulated industrial milling process, exhibit enhanced biomechanical properties. This enhancement is attributed to their superior adaptation to the prepared root canal walls, facilitating the formation of a thicker layer of cement and thereby promoting post retention [13]. The general materials used for the fabrication of a CAD/CAM post are lithium disilicate, zirconia, and resin composite. However, the high modulus of elasticity of posts made from zirconia with 200 GPa and lithium disilicate with 90–110 GPa leads to the transmission of stress into the relatively weaker dentin, which results in root fracture [14]. This is less of a problem now, but with older scanner models and software, optical impressions often failed to detect deep marginal lines on prepared teeth, especially when bleeding was present or when subgingival prosthetic margins were indicated in the aesthetic zone [15]. Whereas the direct digitalization technique is superior to the indirect one, some limitations are present in recording narrow root canal space during post and core scanning, which sometimes is impossible, especially when deep root canals are scanned [16].

The objective of this study is to compare the accuracy of various intraoral scanners employing different acquisition technologies in scanning root canals for CAD/CAM post fabrication. The null hypotheses were formulated as follows: (1) there would be no significant difference among the intraoral scanners used to scan the simulated root canal depth, and (2) there would be no numerical difference between the actual area value of the examined region and the area value recorded by both the intraoral and laboratory scanners.

## 2 Materials and Methods

This study utilized three distinct intraoral scanners (IOSs): the CEREC Primescan (Dentsply Sirona), TRIOS (3Shape), and CEREC Omnicam (Dentsply Sirona), in addition to a desktop scanner, the inEos X5 (Dentsply Sirona). Each of these intraoral scanners utilizes distinct technology for digitizing dental tissues, as outlined in Table 1.

**TABLE 1:** Technical characteristics of the scanners systems used.

Group	Acquisition technology	Powdering	Software version	Manufacturer
Primescan	High‐resolution sensors and short wave light with optical high frequency contrast analysis for Dynamic Deep Scan	No	CEREC 5.1.8	Dentsply Sirona
Trios 3	Confocal microscopy ultrafast optical sectioning	No	TRIOS Design studio 2022.1	3Shape A/S
Omnicam	Active triangulation (multicolor stripe projection)	No	CEREC 5.1.8	Dentsply Sirona
inEos X5	Digital light stripe projection	Yes	inLab 22.2.0	Dentsply Sirona

**FIGURE 1:**
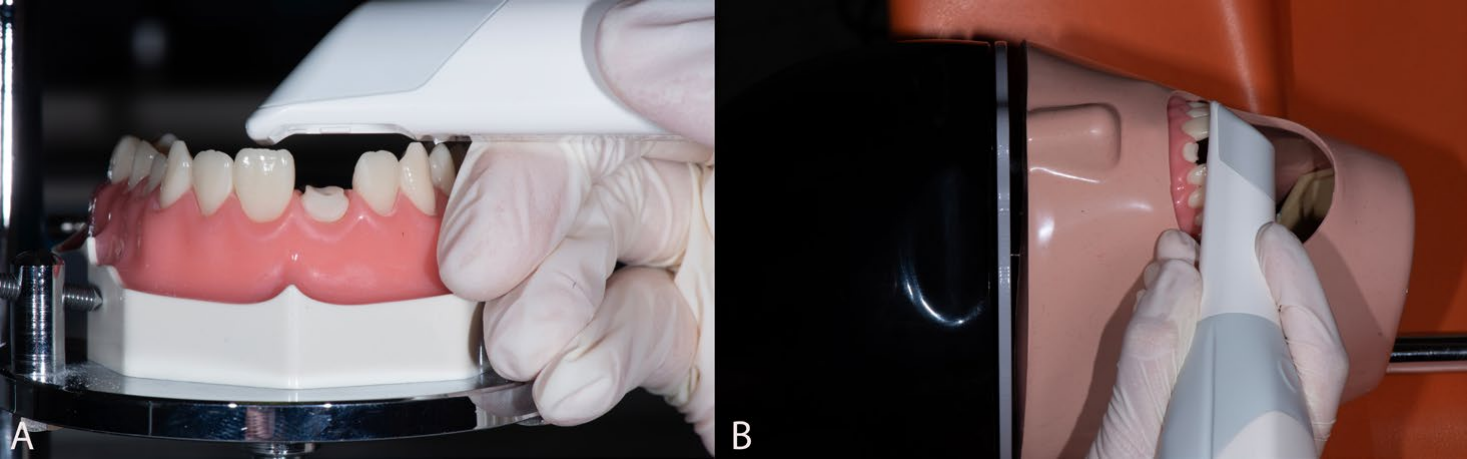
(A) Experimental arrangement involved utilizing a dental simulation mannequin featuring a factory‐made #21 tooth, with a root canal and intact coronal dentin to test the intraoral scanners. (B) In the intraoral simulation, the typodont assembly was securely fastened to a mannequin positioned on a dental chair.

For the experiment, a dental simulation mannequin (P‐Oclusal, Sao Paolo, Brazil) equipped with dental Typodont Jaws (Flex‐Manequim Odontologico, P‐Oclusal), including soft gums and teeth that can be removed and replaced, was selected. On the maxillary typodont, within the anterior left quadrant, the initial central incisor was removed and substituted with a factory‐made #21 tooth. This replacement had a root canal with existing coronal dentin and a fully prepared crown (Figure 1). The size and depth of the prepared root canal were provided by the manufacturer (P‐Oclusal, Sao Paolo, Brazil), the height/depth of the root canal was 10 mm, and the radius was 1.25 mm. Considering that the factory‐produced #21 typodont with root canal surface areas (SAs) was precisely engineered with defined dimensions, the SA of the cone was computed using the formula SA = *πr* ^2^ + *πrl*, where “*r*” represents the radius of the base, “*h*” denotes the height of the cone, and “*l*” represents the slant height, which is the diagonal distance from the apex of the cone to any point along the circular edge of the base. The slant height was determined using the formula *l* = √(*h* ^2^ + *r* ^2^). Consequently, the actual SA in square millimeters (mm^2^) for the root canal region amounted to 40 mm^2^. However, the SA was also verified by micro‐CT (Bruker, Micro‐CT XRM, SkyScan 1276, Nyrecom Software) and it was recorded with a value of 40 mm^2^ and was used as the reference for all comparisons.

According to the scanners used to digitize the maxillary teeth, four groups were formed: Group A with the Primescan scanner, Group B with the Trios 3 scanner, Group C with the Omnicam scanner, and Group D with the inEos X5 desktop scanner. For Group D, the digitization of the maxillary teeth was conducted using the inEos X5 desktop scanner in the same manner as the intraoral scanners used in Groups A, B, and C, ensuring consistency in the digitization process. Group E used an analog digitized impression technique. Polyvinyl siloxane impressions were made, and the resulting physical models were digitized to produce digital datasets. There were two different techniques for the digitization of the maxillary typodont assembly. One approach entailed the utilization of a customized parallelometer (Figure 1A) (Mestra Surveyor, Talleres Mestraitua, SL Mestra, Spain), while the other method involved an intraoral simulation (Figure 1B). Previous studies have demonstrated that different scanning patterns can influence the accuracy of the obtained results from intraoral digital scans [17, 18]. The scanning pattern is the sequence used to capture an intraoral digital scan; generally, one should use the scanning protocol recommended by the manufacturer of the chosen intraoral scanner [19]. During the scanning with the intraoral scanner, the mandibular typodont was first stabilized horizontally and then at multiple angular positions using a custom‐made parallelometer. This custom‐made device provided standardized levels and distances for all the scanning levels and distances with the different intraoral scanners, together with the maxillary typodont assembly. However, teeth #11 and #22 were prevented by the adjacent teeth mesially and distally from being accessed by the cervical region by the intraoral scanner (Figure 1). The scanning methodology was uniform for all scanners. It started with occlusal surface scans of the maxillary left second molar (#27), followed by occlusal surface scans of the maxillary right second molar (#17). The tip was then angulated 45° lingually, tracing the dental arch back to the maxillary left second molar, and finally occlusally swept across the entire arch to return to the start point. To finalize these scans, the scanner was angled at 45° buccally to capture the full arch to the left second molar. The scanning time was recorded using a digital online stopwatch preset for 1 min. A total of 30 consecutive intraoral scans were taken for each specimen according to ISO 20896‐1. The scans were exported in STL format. To simulate the intraoral scanning, the typodont assembly was mounted on a mannequin, which was rigidly attached to the headrest of the dental chair, with the IOS appliances always on the right side of the chair [20]. The scanning sessions were performed by an experienced dentist with over 10 years of active involvement in the application of intraoral scanning and over 1000 restorations scanned, designed, and milled. Each specimen was scanned 30 times. To simulate real clinical conditions, the interincisal opening was fixed at 50 mm while the duration of scanning was limited to 60 s and timed with a digital online stopwatch. The ambient lighting was set consistently to 1000 lx using a luxmeter while all scans were done. The exported scan data were in the original standard tessellation language format (.STL) format without altering anything. A total of 300 STL files were obtained, and the files coming from group D with the inEos X5 laboratory scanner became the standard digital model for visual comparison to the scans obtained by the other intraoral scanners and the digitized analog impression method. To enhance the fidelity of clinical simulation, an analog impression of the dental simulation mannequin was obtained. Consequently, an analog root canal impression of the #21 tooth was conducted using polyvinyl siloxane (PVS) materials of both light body and medium consistencies (Identium medium, Kettenbach SNC, France) alongside a plastic tray. Initially, light body polyvinyl siloxane was injected into the post space, succeeded by the placement of a DT Light‐Post Illusion X‐RO (RTD, France), and subsequently, medium consistency polyvinyl siloxane was applied. Visual inspection of the root canal impression was carried out, with the length of the impression measured using a periodontal probe and file. Additionally, the impression underwent evaluation for indications of serrated RDT post show‐through or other defects. The analog impression was digitized using an inEos X5 laboratory scanner, which provided the reference model for comparisons with the data obtained from the intraoral scanners. The ambient conditions were strictly controlled for all procedures. When the intraoral scanning was performed, the ambient light was held between 1000 lx, measured by a luxmeter, Smart Sensor ST9620 from EMIN Myanmar Co. All IOSs were calibrated as per the manufacturer's instructions before the scanning session started, and after every tenth intraoral scan. After the initial scan with the intraoral scanner Trios 3, the zoom tool was employed to enhance the scanning depth, focusing on the factory‐prepared root canal.

### 2.1 Trueness Evaluation

**FIGURE 2:**
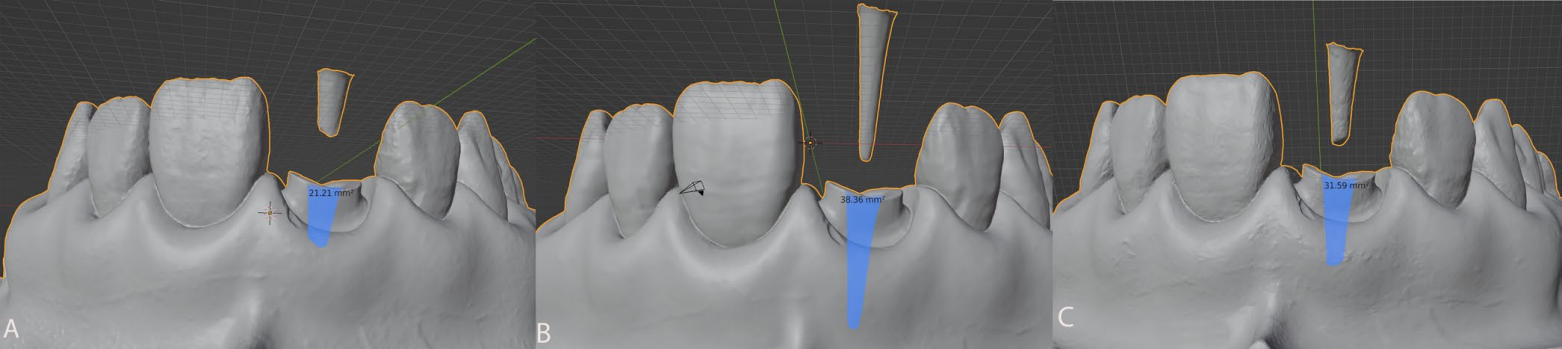
Trueness evaluation and measurement of the root canal surface area were conducted using integrated CAD tools in Blender for STL digital impressions, aiming to assess the geometric precision of the prefabricated root canal. Digital impressions were obtained with (A) Omnicam, (B) Primescan, and (C) Trios 3.

**FIGURE 3:**
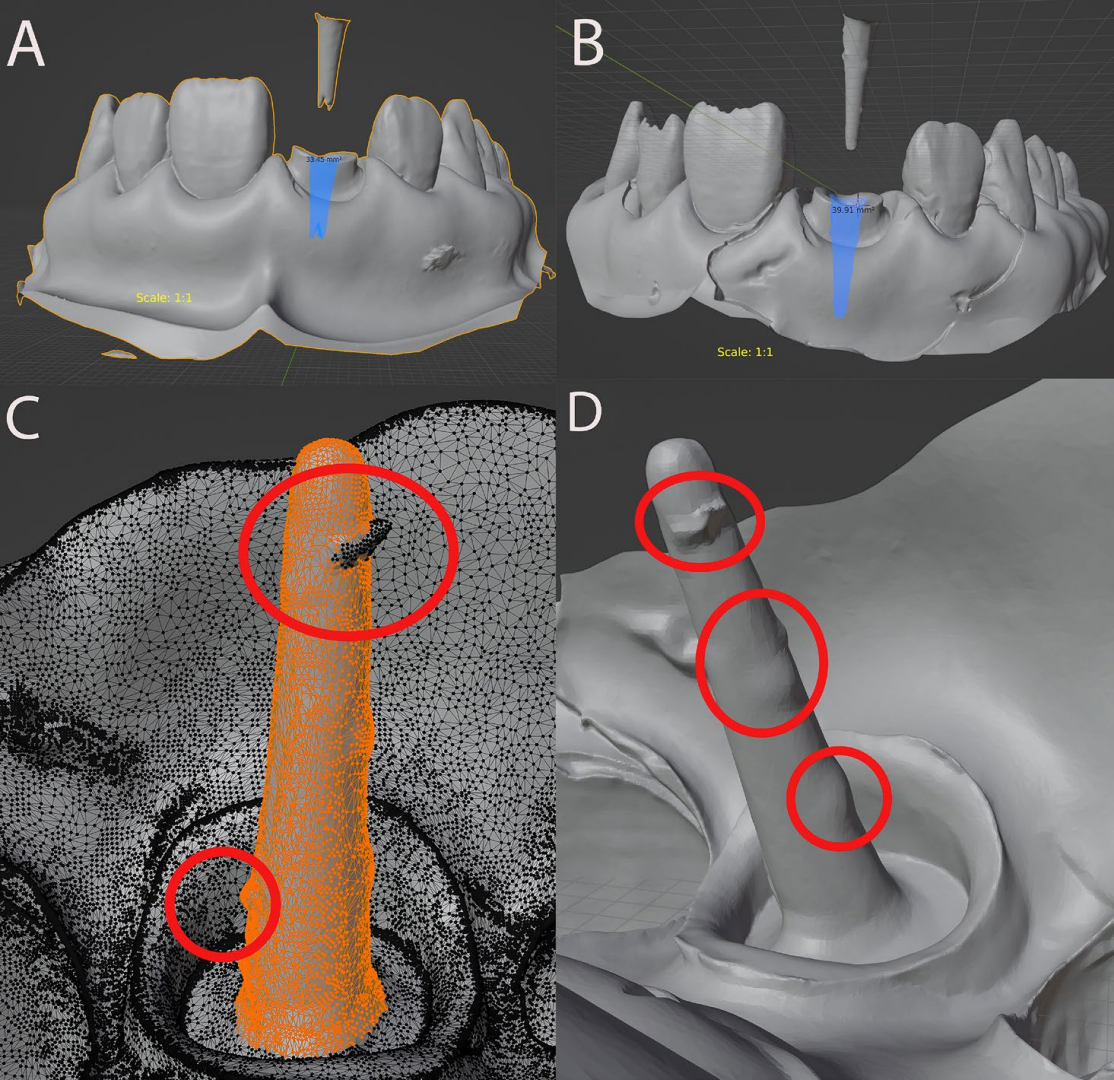
The accuracy and surface area measurements of the root canal were evaluated using integrated CAD tools in Blender for STL digital impressions. This assessment aimed to determine the geometric precision of the prefabricated root canal. (A) Digital impressions acquired with inEos X5, (B) Digitized analog impressions, (C and D) Irregularities in the digitized analog impression material.

Trueness is the proximity of the average test result to the true reference value. To check for trueness, the SA of the root canal in all STL files obtained from CAD software scans was measured. The evaluation of the root canal's SA involved several procedural steps utilizing Blender software (Blender Foundation, Amsterdam, Netherlands). First, the closed geometric STL file was imported into the software environment. In Edit mode, the SA of the root canal was calculated using the integrated measurement tools provided by the software. This approach ensured accurate and consistent determination of the root canal's SA within the digital model. A duplicate of the rectangular tank surface mesh was observed. The entire mesh was selected, and its area in square millimeters was measured using the CAD software measurement tools. Twenty measurements were performed for each mesh. The images produced were representative of comparisons between different intraoral scanners (Omnicam, Primescan, Trios 3, inEos X5 and the digitized analog impression) and were used to visually evaluate the accuracy of post canal SA measurements generated by each scanner. The differences in SA suggest variations in scanner precision when capturing the geometry of the post canal region (Figures 2 and 3).

The STL data from the desktop scanner provided a further visual gold standard with which the accuracy of the experimental subgroups could be compared to the control subgroup. The manufacturer claims that the desktop laboratory scanner achieves trueness of less than 1.3 μm and precision of less than 0.4 μm for standard “inlay” test specimens.

Besides the measurements obtained through the Blender CAD software, trueness was determined for each intraoral scanner by visual examination of mesh pairs for each test subgroup against the reference model (STL file coming from the desktop laboratory scanner). A reverse engineering software package, Geomagic Control X (Artec 3D, Rue Lou Hemmer, Luxembourg), was used to further assess the accuracy of the scan data for the experimental models. The CAD tools of the software utilized a best‐fit algorithm to align each reference scan with the associated experimental scan. Statistical software, G*Power 3.1.9.6 (Heinrich‐Heine‐Universität Düsseldorf, Düsseldorf, Germany), was used to calculate the sample size for the number of STL files and scans. The calculation revealed that 15 samples per group were necessary to achieve 80% power with an effect size of 0.4 at a pre‐set *α* level of *α* = 0.05. However, to comply with ISO 20896‐1 standards, 30 consecutive intraoral scans were performed on each specimen, ensuring greater precision and reliability in measurements.

## 3 Statistical Analysis

The statistical analysis in this study was performed with SPSS (IBM, version 29, IBM Corp., Armonk, NY, USA) in order to evaluate differences in SA across five different scanning methods: Primescan, Trios 3, Omnicam, inEos X5, and the digitized analog impression for differences regarding the measurement of SA. Also, two scanning techniques, the Parallilometer method and the Phantom method, were compared to evaluate their impact on SA measurements. All tests used a pre‐set level of significance of α = 0.05. The normality of the SA data was examined by the Shapiro–Wilk test. The outcomes of the test indicated the violation of normality for some scanners. Since the sample size was sufficient, parametric tests were used based on the robustness of ANOVA in such cases. Two‐way ANOVA was performed to evaluate the main effects of scanner type and scanning method (Parallilometer vs. Phantom) and their interaction on SA measurements. The independent variables were scanner type and scanning method, while the dependent variable was the SA. The following statistical steps were taken: (a) Main effects: Independent evaluation of the effect of scanner type and scanning method on SA. (b) Interaction effects: Assessment of whether the relationship between scanner type and SA depended on the scanning method. (c) Post hoc analysis: Tukey's HSD test was employed to identify specific group differences when variances were equal; Games–Howell was considered if equal variances were not assumed.

## 4 Results

The mean values and standard deviations of the SA (mm^2^) for all intraoral scanners are presented in Table 2 and graphically illustrated in Figure 5. Significant differences in SA among the five scanning systems were found. These findings indicate that the depth of the scanned area significantly influenced scanning accuracy, leading to the rejection of the first null hypothesis. Additionally, the discrepancies observed between the actual SA of the root canal and the scanned SAs obtained from all intraoral scanners resulted in the rejection of the second null hypothesis. A two‐way ANOVA demonstrated that the type of scanner had a highly significant effect (*F* = 1836.736, *p* < 0.001), while the method of scanning (Parallilometer vs. Phantom) did not influence the measurement significantly (*F* = 0.265, *p* = 0.900), indicating that the effect of the scanner on SA measurements was consistent across methods. The highest accuracy related to intraoral scanning, for both methods of impression, was observed with the Primescan scanner (38.86 ± 0.65) for the parallilometer method and the lowest for the Omnicam scanner (21.73 ± 2.71) with the same method. While Primescan and the digitized analog impression had the highest SA measurements, Omnicam consistently had the lowest values. Tukey's HSD post hoc test identified significant differences between several scanners. Primescan had significantly higher SA measurements than Trios 3 (mean difference = 7.6925, *p* < 0.001), Omnicam (mean difference = 16.9870, *p* < 0.001), and inEos X5 (mean difference = 7.8135, *p* < 0.001), but no significant differences with the digitized analog impression (*p* = 0.211). Trios 3 had significantly higher SA measurements than Omnicam (mean difference = 9.2945, *p* < 0.001) but was not significantly different from inEos X5 (*p* = 0.986). Omnicam had significantly lower SA values than all other scanners, including the digitized analog impression (mean difference = 17.4900, *p* < 0.001). To summarize the above significant differences in SA measurements across the scanners, with Primescan and the digitized analog impression measuring the largest SA of the root canal, while Omnicam provided the smallest. The method of scanning (Parallilometer—Phantom) did not influence the measurements significantly. No interaction effect was noted between scanner and method.

**TABLE 2:** Results of the accuracy of the impressions taken: type of scanner, method of impression, average accuracy with standard deviation.

Scanner	Sa (mm^2^)	
	Phantom	Parallilometer
Primescan	38.82 ± 0.67^A,a^	38.86 ± 0.65^A,a^
Trios 3	30.95 ± 1.09^A,b^	31.34 ± 0.93^A,b^
Omnicam	21.73 ± 2.71^A,c^	21.97 ± 0.87^A,c^
inEos X5	31.02 ± 0.02^d,b^	
Analog digitized impression	39.34 ± 0.11^e^	

*Note*: Different superscript capital letters suggest statistically significant differences within different rows. Different superscript lowercase letters suggest statistically significant differences within the same column.

Figure 2 reveals notable differences in the SA measurements of the post canal across the three intraoral scanners, highlighting variations in the precision of each scanner's ability to capture the root canal geometry. The SA calculated by the Omnicam scanner was the smallest of the three, suggesting that it might capture a more restricted or reduced SA, potentially indicating less detailed scanning or a narrower perception of the canal's geometry. In contrast, the Primescan scanner measured a significantly larger SA compared to Omnicam. This implies that Primescan provides a more expansive and detailed view of the post canal, covering a larger area and suggesting higher accuracy or sensitivity to the canal's contours. The Trios 3 scanner measured a SA that falls between those of Omnicam and Primescan. While it captures more detail than Omnicam, it does not reach the extent of the area covered by Primescan, possibly reflecting a medium level of scanning accuracy in depicting the full surface of the post canal. These differences likely reflect the varying abilities of the scanners to accurately depict complex dental structures such as the post canal.

Figure 3 highlights significant SA deviations and irregularities between the desktop laboratory scanner and the digitized analog impression. In the case of the desktop scanner, the smaller measured SA could be due to limited post space. For the digitized analog impression, the larger SA measured is likely a result of the inherent limitations of the silicone material. These inconsistencies may compromise the post's fit and overall stability. The digitized analog impression shows a much more expansive area compared to the previous scans, possibly due to inaccuracies or irregularities introduced by the silicone material, which increase the SA. While the overall structure appears smooth, this larger SA suggests deviations from the true geometry, potentially leading to a poor fit in practice.

The image also highlights mesh distortions (marked in red circles) in the digitized silicone impression. The mesh overlay reveals clear imperfections in the scanned surface, likely contributing to inaccuracies. These irregularities reflect areas where the silicone material deformed or failed to capture fine details, potentially causing deviations in the post fit. The inconsistencies, especially around the midsection and upper portion of the post, further emphasize the limitations of silicone impressions. Such deviations can lead to compromised stability and improper fitting when the post is placed in the canal.

**FIGURE 4:**
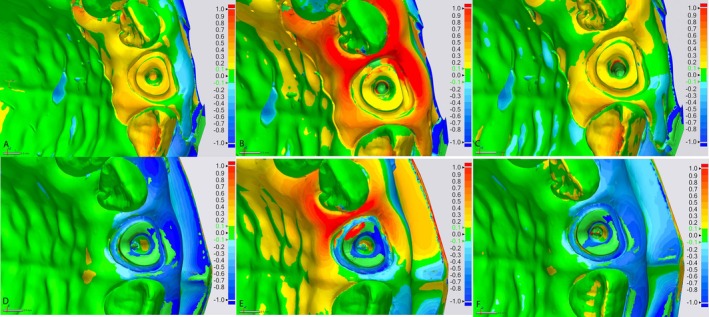
Color map comparisons of intraoral scanners: (A–C) with the digitized analog impression, and (D–F) with the desktop laboratory scanner. (A, D) Primescan, (B, E) Trios 3, and (C, F) Omnicam.

For visual and qualitative analysis of trueness across the different scanner groups, subgroups, and depth areas, Figure 4 includes a color‐coded signed difference between the intraoral scanners and the analog digitized impression. The red color represents areas with significantly higher differences in SA, while green and yellow indicate areas with smaller differences in SA. Figure 6 illustrates images of cross‐sectional 3D‐printed models at the level of an anterior tooth prepared for post placement. The models give a great insight into the depth and contour of the post space, as captured by a specific intraoral scanner. The post canal preparation is clearly visible, indicating the capability of a scanner to record with great accuracy the internal anatomy of the tooth, depth, and shape of the post space, especially for the Primescan scanner. Such cross‐sectional models are helpful in establishing the accuracy of a scanner in capturing critical subgingival details—for instance, the depth of post space—which may be essential in the proper fitting of restorations. The images highlight the scanner's ability to provide sufficient depth information, making it a valuable tool for CAD/CAM workflows in prosthetic dentistry.

**FIGURE 5:**
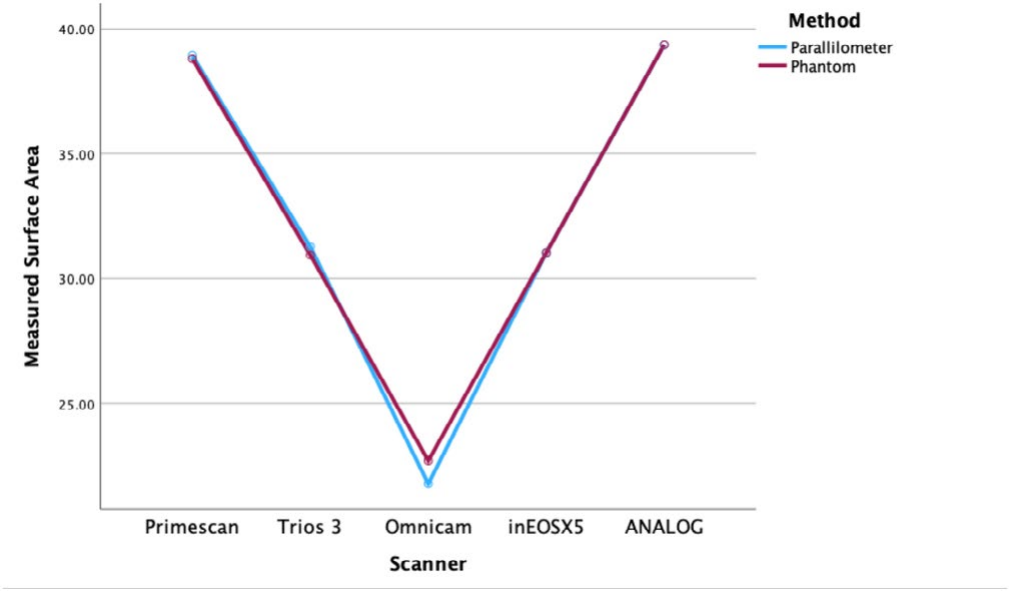
Comparative surface area measurements for five different scanners and one analog technique: Primescan, Trios 3, Omnicam, inEOS X5 and Digitized Analog impression. Y‐axis is the measured surface in mm^2^. Data is contributed for two different methods: Parallilometer method—blue line; Phantom methodred line. Both methods present the same tendencies of the measurements of surface area for the different scanners; the Omnicam recorded the lowest area, and the digitized analog impression method registered the maximum in all cases. Most of the closeness of the lines shows that there is a high agreement in most of the scanners by the two methods with only minor discrepancies, thus indicating high reliability and reproducibility of measurement of subgingival anatomy.

**FIGURE 6:**
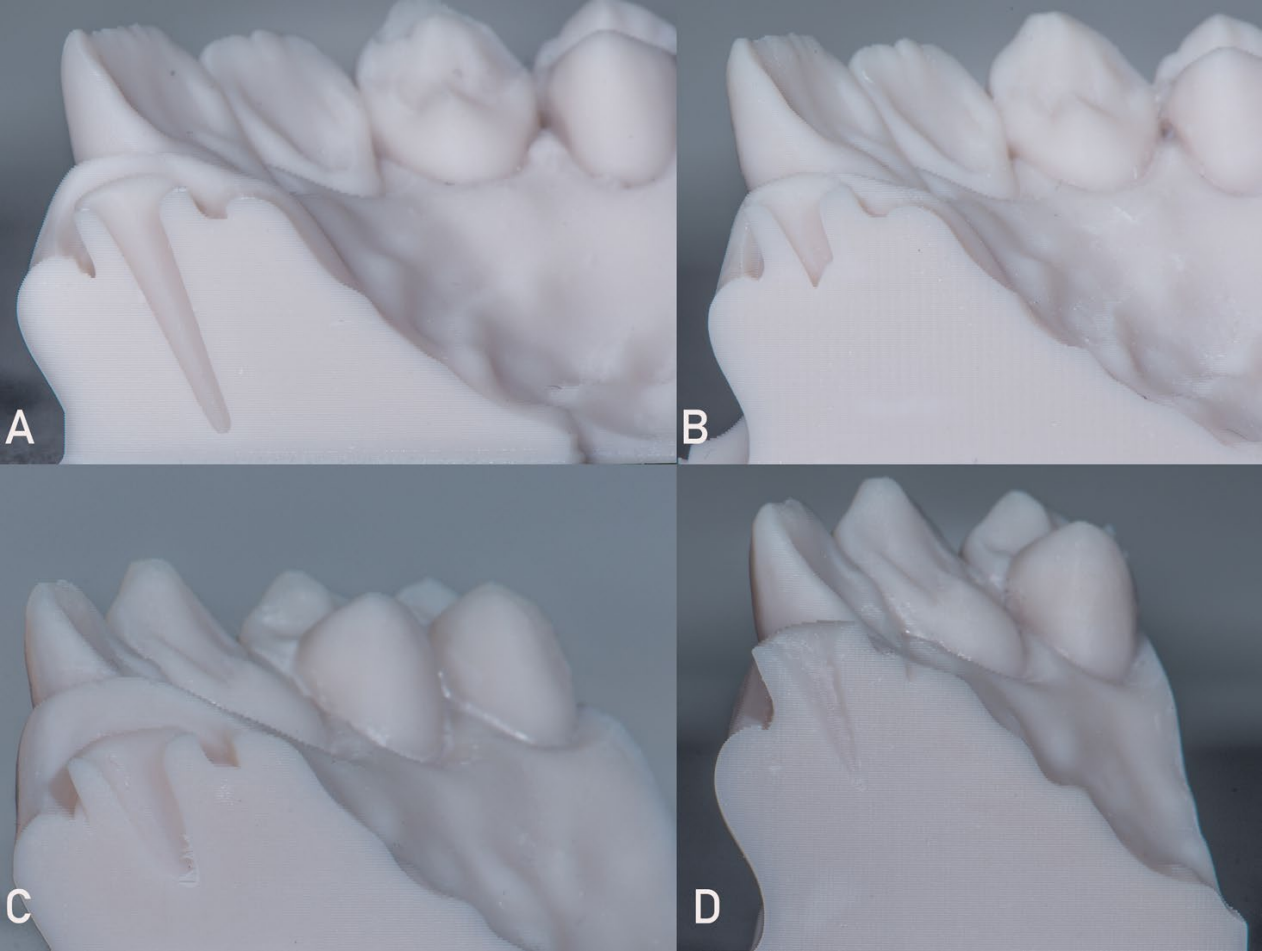
Representative cross‐sections 3D‐printed images demonstrating the intraoral scanner's ability to accurately capture the depth and anatomy of an anterior tooth's post space preparation. (A) Primescan, (B) Omnicam, (C) Trios 3, and (D) inEos X5.

## 5 Discussion

The results of the study demonstrate that the depth of the scanned area has a significant impact on the scanning accuracy of the intraoral scanners examined. Consequently, the first null hypothesis was rejected. Moreover, there was a statistically significant difference between the actual SA of the root canal and the resulting scanned SA from all the intraoral scanners; thus, the second null hypothesis was also rejected.

The necessity of an interradicular post in the rehabilitation of severely damaged endodontically treated or compromised teeth, in order to maintain tooth function and increase survival probability, has been proven successful and has a high survival rate [21, 22]. The customized metal post and cores have been used for many years despite the high elastic modulus, which concentrates a high amount of stress in the surrounding radicular dentin and may result in root fractures [23]. Due to this property of the customized metal post several different approaches or several different materials were used and studied. Gold alloys and silver–palladium were considered superior to base metal alloys but also had a high modulus of elasticity compared to dentin, increasing the risk of root fracture [24]. Zirconia was an attempt to substitute the traditional materials and demonstrated greater fracture resistance in endodontically treated teeth compared to both cast metal posts and cores. Nevertheless, zirconia's high modulus of elasticity transmits elevated stresses to root dentin, thereby heightening the potential for root fractures [25].

The integration of CAD/CAM technology for post and core fabrication was initially detailed in 2007 by two studies [10, 26]. This was succeeded by case reports employing diverse methodologies and materials, as expounded upon in subsequent sections [27, 28] along with some in vitro studies [13]. However, all the studies used indirect methods for the digitization of the prepared root canal, such as using acrylic, wax, or resin patterns to replicate the morphology of the root [10, 11, 12, 13] using scan bodies to capture the dimensions of the post component in the post‐and‐core restoration process [29], manufacturing the CAD/CAM post to match prefabricated posts [1, 27, 30] or impression digitalization [30, 31, 32]. Other studies indicate that employing a direct or fully digital workflow with an intraoral scanner leads to superior adaptation compared to indirect digitalization methods involving patterns or impressions [33]. Although the direct digitalization approach generally offers advantages over the indirect method, challenges may arise in accurately capturing the narrow confines of the root canal space during post and core scanning. It is noteworthy that historically, all the intraoral scanners had limitations in terms of depth of scan due to hardware capabilities [34]. In the past, several intraoral scanners have been reported to be capable of scanning post‐space lengths of up to 8–10 mm. Thus, most studies generally adopt a post‐space preparation length of 5–9 mm before scanning. In cases where the post space is greater than 10 mm, an indirect method of fabricating CAD/CAM post and cores has been resorted to by researchers. However, with advancements in both software and hardware, intraoral scanners demonstrate promising potential for scanning deeper in subgingival areas or potentially into the root canal [35, 36].

Limited research has been conducted to evaluate the effectiveness of intraoral scanners in accurately scanning the depth required for dental restorations [37, 38], the depth of margin location [15, 38, 39] or varying implant depths [40]. However, to the authors' knowledge, no research has compared intraoral impressions of a factory‐prefabricated root canal of a known surface with those obtained from a laboratory scanner for post‐fabrication. In this study, both intraoral and in vitro conditions were simulated to evaluate whether there is a correlation between the accuracy of intraoral scanners and the digital impression of the prefabricated root canal. A high‐trueness extraoral scanner (inEos X5) was utilized as a reference scanner and compared with the intraoral scanners [41, 42]. Additionally, a silicone impression was used to determine if this common method, employed by many dentists, is more accurate than direct digitization of prepared dental tissues using intraoral scanners.

While the study provides valuable insights into the accuracy of intraoral scanners and CAD/CAM technologies, it is important to note that the measurement of the SA serves only as an indication of the accuracy of CAD/CAM‐fabricated posts. The clinical relevance lies in whether posts fabricated by milling or 3D printing offer greater accuracy and clinical performance compared to traditionally fabricated posts. This question extends beyond the scope of the current study and warrants further investigation to determine the relative effectiveness of these fabrication methods in real‐world clinical settings. While the analog impression method initially appears to offer superior accuracy in SA measurements, it is crucial to consider that this high accuracy stems from irregularities inherent to the silicone impression material. These irregularities, although leading to a closer match in SA measurements, may result in a poor fit of the post within the root canal, potentially compromising the restoration's stability and longevity. The analog method's superior accuracy does not necessarily equate to clinical effectiveness due to these limitations. This highlights the need for continued innovation in digital scanner technology to address these challenges and improve clinical outcomes. The major advantage of this in vitro study is that accuracy was measured directly against the actual surface of the prefabricated root canal; thus, it was a standardized surface, rather than relying on reverse engineering software. The latter method necessitates the use of multiple fiducial markers to enhance alignment accuracy between reference scans and sample scans; however, discrepancies between the aligned images can still occur. The reverse engineering software was used to visualize deviation patterns (Figure 4) using color‐coded maps when the reference scan was juxtaposed with intraoral scanner (IOS) scans. A color map was produced for the visual inspection of surface displacements between overlapped digital models. Colored areas in green represent minimal displacements, ±0.1 mm from reference data, while red and blue represent outward and inward displacements of +1.0 and −1.0 mm, respectively. As shown in Figure 4, the comparison between the reference impression and those from the Omnicam and Trios 3 intraoral scanners in the post canal region reveals areas of red and blue. These colors indicate several inward and outward displacements, signifying significant deviations from the reference.

## 6 Conclusion

The study results demonstrate that the depth of the scanned area significantly impacts the accuracy of intraoral scanners, with the analog impression method exhibiting superior SA accuracy. However, digital methods, particularly those using intraoral scanners such as the CEREC Primescan, offer commendable accuracy and significant advantages in CAD/CAM integration and efficiency. Continued advancements in digital scanner technology are necessary to overcome current limitations and further improve accuracy for clinical applications. Intraoral scanners, particularly the CEREC Primescan, demonstrated commendable accuracy; yet in some groups, challenges remain in effectively capturing the complex geometry of deep root canals. Despite these challenges, digital scanning methods offer significant advantages in terms of efficiency and integration with CAD/CAM workflows, which are essential for modern dental practices aiming to streamline procedures and reduce patient chair time. While the analog method demonstrated superior SA accuracy, it was less practical and posed limitations due to inherent material irregularities that could result in poor post fit. Thus, the analog technique showed superior accuracy in specific metrics, but digital methods excelled in practicality and clinical workflow integration. This underscores the necessity for continued improvements in digital scanner technology to enhance their precision in capturing intricate root canal details. Integrating the precision of analog impressions with the efficiency of digital techniques could potentially yield optimal outcomes for post‐and‐core restorations. Ultimately, this study emphasizes the need for ongoing advancements in scanner technology to address the limitations identified and to further refine the accuracy of digital impressions, thereby improving clinical outcomes and patient satisfaction.

## Conflicts of Interest

The authors declare no conflicts of interest.

## Data Availability

The data that support the findings of this study are available from the corresponding author upon reasonable request.
